# Supplementary material for: FERN – a Java framework for stochastic simulation and evaluation of reaction networks
Source: BMC Bioinformatics. 2008 Aug 29;9:356. doi: 10.1186/1471-2105-9-356 (PMC2553347; doi:10.1186/1471-2105-9-356)
Supplement: Additional file 1 — FERN distribution, Version 1.3. This archive contains the FERN source code and binaries as well as documentation and example models in FernML and SBML. [file 1471-2105-9-356-S1.zip › fern/doc/javadoc/index-files/index-1.html]

A-Index


---


|  |  |  |
| --- | --- | --- |
| **Overview** \| Package \| Class \| Use \| **Tree** \| **Deprecated** \| **Index** \| **Help** | |  |
| PREV LETTER   **NEXT LETTER** | **FRAMES**    **NO FRAMES**     **All Classes** |  |


A B C D E F G H I J L M N O P R S T U V 

---


## **A**

**a** - Variable in class fern.simulation.Simulator: Contains the propensities of the reactions. **a\_sum** - Variable in class fern.simulation.algorithm.GillespieEnhanced: **a\_sum** - Variable in class fern.simulation.algorithm.HybridMaximalTimeStep: **AbstractBaseTauLeaping** - Class in fern.simulation.algorithm: Base class for all tau leaping procedures (which are different in the methods choosing the timestep candidates for critical and noncritical reactions). **AbstractBaseTauLeaping(Network)** - Constructor for class fern.simulation.algorithm.AbstractBaseTauLeaping: Create the simulator for a given network. **AbstractKineticConstantPropensityCalculator** - Class in fern.network: Base implementation of a `PropensityCalculator`. **AbstractKineticConstantPropensityCalculator(int[][])** - Constructor for class fern.network.AbstractKineticConstantPropensityCalculator: Creates a `AbstractKineticConstantPropensityCalculator` by an array of adjacency arrays for the reaction's reactant species (which are the only one needed for the propensity calculation). **AbstractNetworkImpl** - Class in fern.network: Base implementation for the `Network` interface. **AbstractNetworkImpl(String)** - Constructor for class fern.network.AbstractNetworkImpl: Create the network and give it an identifier. **AbstractTauLeapingPropensityBoundSimulator** - Class in fern.simulation.algorithm: There are some possibilities to bind the expected change of the propensities by a value epsilon in order to fulfill the leap condition. **AbstractTauLeapingPropensityBoundSimulator(Network)** - Constructor for class fern.simulation.algorithm.AbstractTauLeapingPropensityBoundSimulator: **actionPerformed(ActionEvent)** - Method in class fern.cytoscape.CytoscapeVisualizer.ShowMainFrameAction: **actionPerformed(ActionEvent)** - Method in class fern.cytoscape.CytoscapeVisualizer.SimulationAction: **activateReaction(int, double, Simulator.FireType, int)** - Method in class fern.cytoscape.CytoscapeColorChangeObserver: **activateReaction(int, double, Simulator.FireType, int)** - Method in class fern.example.CellGrowthObserver: **activateReaction(int, double, Simulator.FireType, int)** - Method in class fern.network.sbml.SBMLEventHandlerObserver: **activateReaction(int, double, Simulator.FireType, int)** - Method in class fern.simulation.observer.AmountAtMomentObserver: Do nothing **activateReaction(int, double, Simulator.FireType, int)** - Method in class fern.simulation.observer.FireTypeObserver: Counts the firing for the given `Simulator.FireType` **activateReaction(int, double, Simulator.FireType, int)** - Method in class fern.simulation.observer.InstantOutputObserver: Prints event. **activateReaction(int, double, Simulator.FireType, int)** - Method in class fern.simulation.observer.IntervalObserver: Do nothing. **activateReaction(int, double, Simulator.FireType, int)** - Method in class fern.simulation.observer.LeapObserver: Do nothing. **activateReaction(int, double, Simulator.FireType, int)** - Method in class fern.simulation.observer.Observer: Gets called before a reaction fires. **activateReaction(int, double, Simulator.FireType, int)** - Method in class fern.simulation.observer.RandomNumberGeneratorCallObserver: Do nothing. **activateReaction(int, double, Simulator.FireType, int)** - Method in class fern.simulation.observer.ReactionIntervalObserver: Record the firing of mu, if mu is registered. **add(int)** - Method in interface fern.analysis.IntSearchStructure: **addAxes(Axes)** - Method in class fern.tools.gnuplot.Axes: Attaches an `Axes` object to this one. **addCommand(String...)** - Method in class fern.tools.gnuplot.GnuPlot: Adds commands to the list of commands which are given to gnuplot by invoking `plot` **addData(double[])** - Method in class fern.benchmark.Benchmark: Adds benchmark data to the data pool. **addData(Collection, String[], String[])** - Method in class fern.tools.gnuplot.GnuPlot: Adds data in order to plot it. **addData(Axes)** - Method in class fern.tools.gnuplot.GnuPlot: Adds data in order to plot it. **addData(Object, String[], String[])** - Method in class fern.tools.gnuplot.GnuPlot: Adds data in order to plot it. **addData(Object[], boolean, String[], String[])** - Method in class fern.tools.gnuplot.GnuPlot: Adds data in order to plot it. **addObserver(Observer)** - Method in class fern.simulation.Simulator: Adds an observer to the list of observers. **addPluginMenu()** - Method in class fern.cellDesigner.FernCellDesignerPlugin: **addTypesToFileChooser(JFileChooser)** - Static method in class fern.network.NetworkLoader: Adds available types of networks to a `JFileChooser`. **adjListAsPro** - Variable in class fern.analysis.AnalysisBase: Contains the adjacency list for molecule species towards reactions, where the species is a product. **adjListAsRea** - Variable in class fern.analysis.AnalysisBase: Contains the adjacency list for molecule species towards reactions, where the species is a reactant. **adjListPro** - Variable in class fern.network.AbstractNetworkImpl: Stores the adjacency lists of reactions to their products. **adjListRea** - Variable in class fern.network.AbstractNetworkImpl: Stores the adjacency lists of reactions to their reactants. **AmountAtMomentObserver** - Class in fern.simulation.observer: Observes the amount of some molecule species at a certain moment. **AmountAtMomentObserver(Simulator, double, String...)** - Constructor for class fern.simulation.observer.AmountAtMomentObserver: Creates the observer for the given simulator, the given theta and the given species **AmountIntervalObserver** - Class in fern.simulation.observer: Observes amounts of molecule species repeatedly after certain intervals. **AmountIntervalObserver(Simulator, double, int...)** - Constructor for class fern.simulation.observer.AmountIntervalObserver: Creates the observer for a given simulator, a given interval and given species indices **AmountIntervalObserver(Simulator, double, String...)** - Constructor for class fern.simulation.observer.AmountIntervalObserver: Creates the observer for a given simulator, a given interval and given species names. **amountManager** - Variable in class fern.network.AbstractNetworkImpl: Stores the `AmountManager` of the network. **AmountManager** - Class in fern.network: The `AmountManager` is one of the most important connections between a `Network` and a `Simulator`. **AmountManager(Network)** - Constructor for class fern.network.AmountManager: Creates an `AmountManager` for a given network **AnalysisBase** - Class in fern.analysis: This class can be used as a base class for many analysis algorithms (like AutocatalticNetworkDetection, ShortestPath, ...). **AnalysisBase(Network)** - Constructor for class fern.analysis.AnalysisBase: Creates an analysis instance. **AndController** - Class in fern.simulation.controller: Implements a `SimulationController` composed of many `SimulationController`. **AndController(SimulationController...)** - Constructor for class fern.simulation.controller.AndController: **annotate(String, String)** - Method in class fern.analysis.AutocatalyticNetworkDetection: Adds annotations to each autocatalytic reaction / species. **annotationManager** - Variable in class fern.network.AbstractNetworkImpl: Stores the `AnnotationManager` of the network. **AnnotationManager** - Interface in fern.network: AnnotationManager objects are the places, where static (or quasi-static) properties of a network are stored. **AnnotationManagerImpl** - Class in fern.network: Base implementation of the `AnnotationManager` interface. **AnnotationManagerImpl()** - Constructor for class fern.network.AnnotationManagerImpl: **applyDefaultStyle(String)** - Method in class fern.tools.gnuplot.Axes: Sets the style of each column to the default style except for columns that already have a style. **applyLabelFormat(String[])** - Method in class fern.simulation.observer.Observer: Applies the actual label format to the whole array (but does not change the passed array, it return a new one) **areEqual(Network, Network)** - Static method in class fern.tools.NetworkTools: Gets whether or not the two given networks contain the same species, the same reactions and yield the same propensities when the amounts of each reactant are respectively 1, 5, 10, 100. **argMax(Map<Integer, Integer>)** - Static method in class fern.tools.NumberTools: Gets the argmax of the given map. **array** - Variable in class fern.tools.gnuplot.ArrayMatrixAxes: Contains the matrix array **ArrayKineticConstantPropensityCalculator** - Class in fern.network: Implementation of an `AbstractKineticConstantPropensityCalculator` which uses an array to store the constants for each reaction. **ArrayKineticConstantPropensityCalculator(int[][], double[])** - Constructor for class fern.network.ArrayKineticConstantPropensityCalculator: Create the propensity calculator with given constants and given reactant adjacency arrays **ArrayMatrixAxes** - Class in fern.tools.gnuplot: Implementation of `Axes` to use with array matrixes of any number type (the type of the arrays is inferred by using the reflection api). **ArrayMatrixAxes(Object)** - Constructor for class fern.tools.gnuplot.ArrayMatrixAxes: Create a new `Axes` object containing the given array matrix without labels / styles. **ArrayMatrixAxes(Object, String[], String[])** - Constructor for class fern.tools.gnuplot.ArrayMatrixAxes: Create a new `Axes` object containing the given array matrix with the given labels /styles (either can be `null`). **AstNodeType** - Variable in class fern.network.sbml.MathTree.InnerNode: **AutocatalyticNetwork** - Class in fern.network.creation: Evolve an autocatalytic network. **AutocatalyticNetwork(char[], Probability, Probability, int)** - Constructor for class fern.network.creation.AutocatalyticNetwork: Creates the autocatalytic network from given monomers, reaction probability, catalysis probability up to a given polymer length. **AutocatalyticNetwork(char[], Probability, Probability, int, boolean)** - Constructor for class fern.network.creation.AutocatalyticNetwork: Creates the autocatalytic network from given monomers, reaction probability, catalysis probability up to a given polymer length. useDefault should usually set to true unless you want to evolve really huge networks. **AutocatalyticNetworkDetection** - Class in fern.analysis: Detects the autocatalytic set of the given network if there is any. **AutocatalyticNetworkDetection(Network)** - Constructor for class fern.analysis.AutocatalyticNetworkDetection: Creates the AutocatalyticDetection by using the in the network built in `CatalystIterator`. **AutocatalyticNetworkDetection(Network, CatalystIterator)** - Constructor for class fern.analysis.AutocatalyticNetworkDetection: Creates the AutocatalyticDetection by using the second argument as `CatalystIterator`. **AutocatalyticNetworkExample** - Class in fern.example: Here, the evolution of a reaction network as proposed by [1] is performed. **AutocatalyticNetworkExample()** - Constructor for class fern.example.AutocatalyticNetworkExample: **avg(double[])** - Static method in class fern.tools.NumberTools: Gets the avg of the array **Axes** - Class in fern.tools.gnuplot: Contains the data columns for `GnuPlot`. **Axes()** - Constructor for class fern.tools.gnuplot.Axes: **axes** - Variable in class fern.tools.gnuplot.GnuPlot

---


|  |  |  |
| --- | --- | --- |
| **Overview** \| Package \| Class \| Use \| **Tree** \| **Deprecated** \| **Index** \| **Help** | |  |
| PREV LETTER   **NEXT LETTER** | **FRAMES**    **NO FRAMES**     **All Classes** |  |


A B C D E F G H I J L M N O P R S T U V 

---
